# Recent Progress in Self-Powered Skin Sensors

**DOI:** 10.3390/s19122763

**Published:** 2019-06-19

**Authors:** Jihong Rao, Zetong Chen, Danna Zhao, Yajiang Yin, Xiaofeng Wang, Fang Yi

**Affiliations:** 1 School of Materials Science and Engineering, Sun Yat-sen University, Guangzhou 510275, China; jihongrao@163.com (J.R.); chenzt22@163.com (Z.C.); zhaodn3@mail2.sysu.edu.cn (D.Z.); 2 Research Institute of Tsinghua, Pearl River Delta, Building B10, Corporation Accelerator, No.11 Kaiyuan Road, Science City, Guangzhou 510530, China; yinyajiang@mail.tsinghua.edu.cn; 3 Guangzhou Grower-Tsingron Energy Co., Ltd., Building B10, Corporation Accelerator, No.11 Kaiyuan Road, Science City, Guangzhou 510530, China

**Keywords:** self-powered, skin sensor, piezoelectric nanogenerator, triboelectric nanogenerator

## Abstract

Self-powered skin sensors have attracted significant attention in recent years due to their great potential in medical care, robotics, prosthetics, and sports. More importantly, self-powered skin sensors do not need any energy-supply components like batteries, which allows them to work sustainably and saves them the trouble of replacement of batteries. The self-powered skin sensors are mainly based on energy harvesters, with the device itself generating electrical signals when triggered by the detected stimulus or analyte, such as body motion, touch/pressure, acoustic sound, and chemicals in sweat. Herein, the recent research achievements of self-powered skin sensors are comprehensively and systematically reviewed. According to the different monitoring signals, the self-powered skin sensors are summarized and discussed with a focus on the working mechanism, device structure, and the sensing principle. Based on the recent progress, the key challenges that exist and the opportunities that lie ahead are also discussed.

## 1. Introduction

Information, energy, and materials are the three pillars of the development of modern human society, among which information technology is the main driving force for development in recent decades. The collection and exchange of different information depends on sensors with different functions, which makes sensors play a vital role in many fields [1,2,3,4,5]. In the fields of health care, medical treatment, sports, human–machine interaction, and so on, sensors are required to obtain various signals from the human body. Obtaining information from the skin—the largest organ of human body in direct contact with the outside world—has always been a vital means in these fields. Skin sensors, as the kinds of devices that can be attached to human skin and collect signals from skin or detect external stimulation from around the environment, can well meet the above needs [6,7,8,9,10,11]. Under the current technological background, a majority of sensors need to be driven by external power sources and cannot work independently and sustainably, which has become one of the main factors restricting the development of skin sensors. One possible solution to address this challenge is to combine skin sensors with energy harvesting and storage components, which provides a feasible scheme for the sustainable operation of skin sensors [12,13,14,15]. Another way to effectively address such a challenge is to develop self-powered skin sensors. Physical movements, the heat emission of the human body, and human secretions are all sources of energy that can be converted into electrical outputs by energy harvesters, and the wave forms of their electrical signals reflect information of such energy sources [16,17,18,19,20,21,22,23]. Several technologies can convert these energy sources into electricity, including the piezoelectric effect, the triboelectric effect, the thermoelectric effect, and the spontaneous redox reaction [24,25,26,27,28,29,30,31]. Based on these principles, various energy harvesters with multiple functions have been developed. Among them, piezoelectric nanogenerators (PENGs) and triboelectric nanogenerators (TENGs) are mechanical energy harvesters that were first proposed in 2006 and 2012, respectively [32,33]. They can harvest mechanical energy from the human body and also work as self-powered skin sensors to detect body motion, touch/pressure, and acoustic sound [34,35,36,37,38,39,40,41,42,43,44]. In addition, non-motion-based energy harvesters, such as thermoelectric nanogenerators and biofuel cells, can serve as self-powered temperature and sweat skin sensors, respectively [45,46,47,48]. The use of new materials enables these devices to have good transparency, portability, flexibility, light weight, comfortability, and biocompatibility. All these advantages above have made self-powered skin sensors receive extensive attention in recent years, and they are undergoing fast development. Hence there is a great need to comprehensively review the recent progress of self-powered skin sensors.

In this review, the recent advances of self-powered skin sensors will be comprehensively reviewed. The self-powered skin sensors will be classified according to the different types of monitoring signals, with a focus on the working mechanism, device structure, and the sensing principle. Section 2, Section 3 and Section 4 will review the self-powered skin sensors for detecting body motion, touch/pressure, and acoustic sound, respectively, which are all related to motion and based on PENGs and TENGs. Section 5 will review other types of self-powered skin sensors, including those used for sensing of body temperature and sweat. The working principle, device structure, device performance, and advantages and disadvantages of different types of self-powered skin sensors will be summarized. Finally, the challenges faced by self-powered skin sensors and the future prospects will be discussed.

## 2. Self-Powered Skin Sensors for Detecting Body Motion

Sensing body motion has a wide range of applications, such as automation, human–machine interaction, medical health, and sports. Current self-powered skin sensors to detect body motion can be mainly divided into two categories according to their working principles. One is based on TENGs and the other is based on PENGs. When mounted on the arms or legs, these sensors can detect human activities such as walking and jogging; when mounted onto fingers, wrists, elbows, or knees, they can monitor joint motion like the bending angle and frequency of joints [35,36,49,50,51,52,53,54,55]. In addition, the skin sensors for detecting body motion are also able to act as a breathing sensor to monitor people’s breathing state when placed on the chest [36,56].

### 2.1. Triboelectric Nanogenerators as Self-Powered Body Motion Skin Sensors

The basic working mechanism of skin TENGs as a body motion sensor is that body motion triggers relative displacement between the two triboelectric parts, which results in potential difference between the two working electrodes and drives electrons to flow across. The features, such as simple structure, easy fabrication, various working modes, multiple options regarding materials, high power density, and good flexibility, all make TENGs a good choice for self-powered skin sensors.

**Figure 1:**
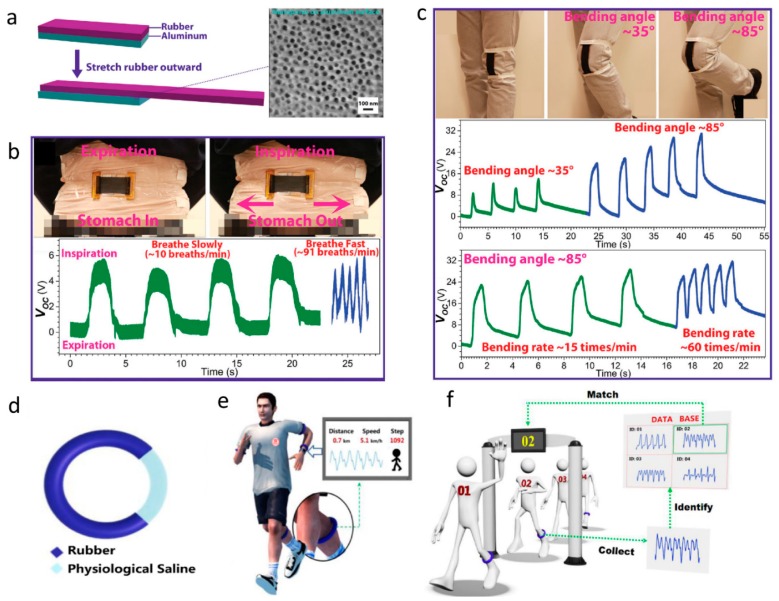
(**a**) Device structure of the stretchable-rubber-based triboelectric nanogenerator (TENG). (**b**) Images and electrical outputs of the TENG on the abdomen during expiration and inspiration. (**c**) Optical images of the device on the knee at different bending angles and voltage responses when bending the knee at different angles and different rates [35]. Copyright 2015, Wiley. (**d**) Typical device structure of the TENG band. (**e**) Schematic illustration of the skin TENG band for body motion detection. (**f**) Schematic illustration of the skin TENG band for identification [49]. Copyright 2018, Elsevier.

**Figure 2:**
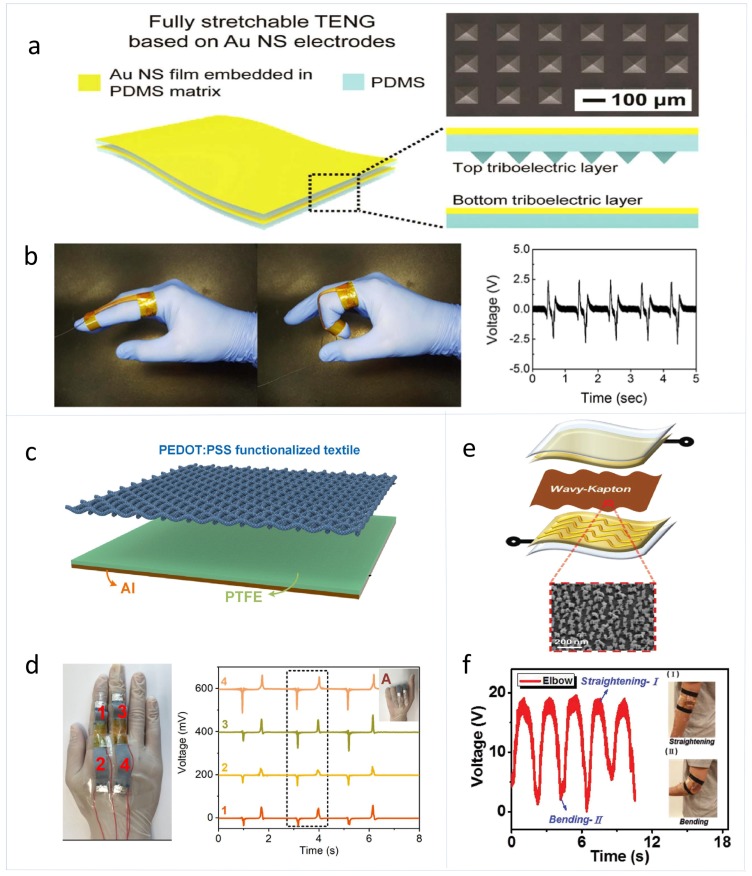
(**a**) Schematic illustration of the structure of the stretchable TENG based on the gold-nanosheet (NS) electrodes. (**b**) Photographs and output-voltage responses to the repeated bending/relaxation of the TENG when installed on the index finger [50]. Copyright 2017, Elsevier. (**c**) Schematic diagram of the structure of PEDOT:PSS functionalized textile based TENG. (**d**) The photographs of the two-arch finger bending sensors installed on the index finger and the middle finger, and output voltages from the four arches when the hand repeats the gestures represented by ‘A’ [51]. Copyright 2018, Elsevier. (**e**) Device structure of the wavy-FTENG. (**f**) Relative changes in voltage versus time for monitoring elbow motion [52]. Copyright 2015, Wiley.

According to the triboelectric materials utilized, self-powered body motion skin sensors based on TENGs can be divided into several categories. The first kind is based on stretchable materials, such as rubber and silicone elastomers. Yi et al. reported a stretchable-rubber-based (SR-based) TENG which utilizes the triboelectricity between a stretchable rubber and an aluminum (Al) film, as shown in Figure 1a–c [35]. The SR-based TENG has a unique working principle. It uses the stretching rather than the position shift of rubber to induce in-plane charge separation between rubber and Al, which leads to the potential difference between the Al electrode and the ground. This SR-based TENG has a short-circuit current density of $7.5{\;\mu A\;m}^{-2}$ and an output power density of $76.27{\;W\;m}^{-2}$. Moreover, this SR-based TENG is able to detect the rates of diaphragmatic breathing and joint movements of a human body like the bending angle and frequency of the knee. Han et al. reported a soft and stretchable triboelectric band consisting of a rubber tube filled with physiological saline, as shown in Figure 1d–f [49]. When worn on different parts of the body, the band can detect changes in muscle volume during movements and can monitor six different types of human motion including swallowing, calf raising, jumping, squatting, breathing, and bicep curling. Furthermore, based on the unique gait patterns of different individuals, this band can be effectively used for identity recognition. Lim et al. fabricated a stretchable and durable TENG based on gold nanosheet embedded electrodes, as shown in Figure 2a,b [50]. With gold nanosheets embedded in PDMS matrix and micro-pyramid patterned PDMS, this type of electrode has excellent mechanical flexibility, tensile properties, and excellent output stability. The TENG can be mounted on the skin to detect the process of repeated bending and relaxation of joints.

The second kind is based on textiles. He et al. reported a skin TENG based on a textile coated by a layer of PEDOT:PSS, which has a maximum output power of 3.26 mW and a power density of $2\;{W\;m}^{-2}$, as shown in Figure 2c,d [51]. This textile-based skin TENG can be applied to capture hand motion and detect finger bending angle. Zhu et al. reported a cotton-sock-based self-powered hybrid skin sensor with PEDOT:PSS coated fabric and PTFE film as the two triboelectric materials [54]. The device has a triboelectric power density of $11\;\mu W/{\mathrm{cm}}^{2}$. This smart sock can realize multiple monitoring, including walking pattern recognition, motion tracking, and gait sensing. The third kind is based on flexible thin films. Yang et al. developed a skin TENG fabricated by assembling serpentine-patterned electrodes and a wavy-structured Kapton film which has an output power density of $5\;{W\;m}^{-2}$, as shown in Figure 2e,f [52]. The advantage of this skin TENG is to work stably under tensile strain or on a curved surface, at both the compressive and stretching mode. It can be attached onto human skin to detect motion of joints and muscles.

### 2.2. Piezoelectric Nanogenerators as Self-Powered Body Motion Skin Sensors

The piezoelectric effect of the materials used in PENGs can polarize the charge due to mechanical stimulation, and then generate electrical signals. PENGs have the advantages of high sensitivity, real-time sensing, and good flexibility, and can be used as self-powered skin sensors by using flexible materials. The reported skin PENGs are mostly based on conventional piezoelectric materials including zinc oxide (ZnO) nanowires, PVDF nanofibers, and lead zirconate titanate (PZT) powders [36,53,57,58,59,60,61,62]. In addition, some materials which have a piezoelectric effect at the nano scale but not in a bulk state have also been used in skin PENGs, such as boron nitride (BN) [63,64].

**Figure 3:**
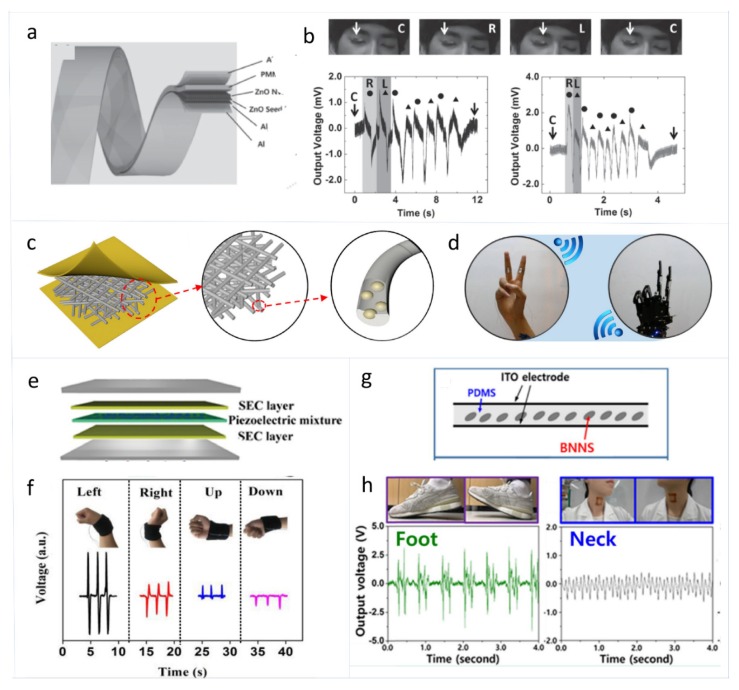
(**a**) Schematic illustration of the structure of super-flexible PENG. (**b**) The PENG attached to the right eyelid as an active sensor for detecting the motion of a human eye ball. Output voltage measured under slow and rapid eye movement [36]. Copyright 2015, Wiley. (**c**) The structure design of the cowpea-structured PVDF/ZnO nanofibers based PENG. (**d**) The application of robot hand control based on the skin PENG [53]. Copyright 2018, Elsevier. (**e**) Schematic illustration of the device structure of the PENG. (**f**) The output voltage of the PENG working as a “smart wristband” under different wrist gestures [60]. Copyright 2018, Elsevier. (**g**) Schematic diagram of the structure of the boron nitride nanosheet based piezoelectric sensor. (**h**) Open-circuit voltage of the device during foot and neck motions [63]. Copyright 2018, Elsevier.

Among the traditional piezoelectric materials, ZnO nanowires have high sensitivity, while PVDF has relatively low sensitivity but good flexibility. Lee et al. reported a kind of skin PENG based on ZnO nanowires as a self-powered sensor to track tiny skin deformation, as shown in Figure 3a,b [36]. On the Al foil, anodic aluminum oxide (AAO) was grown to serve as the insulating layer between the Al electrode and the ZnO nanowire film to block electron transport. This skin PENG can track eye ball motion when attached to an eyelid. Deng at el. fabricated a PENG-based self-powered body motion skin sensor based on cowpea-structured PVDF/ZnO nanofibers which has a sandwich structure by electrospinning, as shown in Figure 3c,d [53]. Due to the synergistic piezoelectric effects of ZnO and PVDF, as well as the good flexibility of the polymer, the skin PENG has a bending sensitivity of $4.4{\;\mathrm{mV}\;\deg}^{-1}$, and can quantitatively measure the bending angle in a range from 44° to 122°. Placing this skin PENG on the joint of five fingers to capture human gestures and to transmit signals to the robotic palm can realize a timely self-powered human–machine interaction system.

Skin PENGs to detect body motion based on piezoelectric ceramics and other materials have also been developed. Chou et al. reported a filler-elastomer-based stretchable PENG developed by incorporating high weight compositions of PZT particles and Ag-coated glass microspheres fillers into the solid-state silicone rubber, as shown in Figure 3e,f [60]. The PENG shows an output power density of 3.93 ${\mu W\;\mathrm{cm}}^{-3}$. This PENG can not only be mounted on the joint to capture the joint posture, but also to monitor the dynamic motion. Kim et al. reported a transparent and flexible piezoelectric sensor based on BN nanosheets which has an output power of 40 μW and a power density of 106 ${\mu W\;\mathrm{cm}}^{-3}$. The skin PENG based on BN nanosheets (1 wt%) dispersed into PDMS as the active layer can work under both tensile stress and compressive stress, as shown in Figure 3g,h [63]. When attached directly to human skin, this PENG can detect the movements of the human foot, neck, wrist, and knee.

Currently, self-powered motion skin sensors are mainly divided into TENG-based and PENG-based ones, which have their own advantages and disadvantages. Table 1 shows the comparison of self-powered skin sensors for detecting body motion in terms of mechanism, materials, motion detected, and output power density. TENG-based self-powered motion skin sensors have a simple fabrication process, multiple choice of materials, and high power density, but their performance is generally affected by environmental factors such as humidity. PENG-based self-powered motion skin sensors have high sensitivity and less environmental impact. However, materials used in these kinds of devices are limited, which must have the piezoelectric effect.

## 3. Self-Powered Skin Sensors for Detecting Touch/Pressure

Human activities induce pressure, and physiological activity in different parts of the body, such as human voice, blood pressure wave, heartbeat, venous pulse, and respiratory movement, and these produce different pressures, which have a wide range from low pressure to high pressure (from 10 to 100 kPa) [65]. Detection of these pressures by pressure sensors is of great significance for the diagnosis and monitoring of various diseases of the human vocal cords, heart, respiratory system, and cardiovascular system [66]. At present, self-powered pressure and touch skin sensors are mainly based on TENGs and PENGs.

### 3.1. Triboelectric Nanogenerators as Self-Powered Touch/Pressure Skin Sensors

TENGs as self-powered touch/pressure skin sensors are mainly working in two kinds of modes: single-electrode mode and attached-electrode mode. The similarity between them is that the touch/pressure induced motion is in the vertical direction, and so is the contact/separation of the two triboelectric parts.

**Figure 4:**
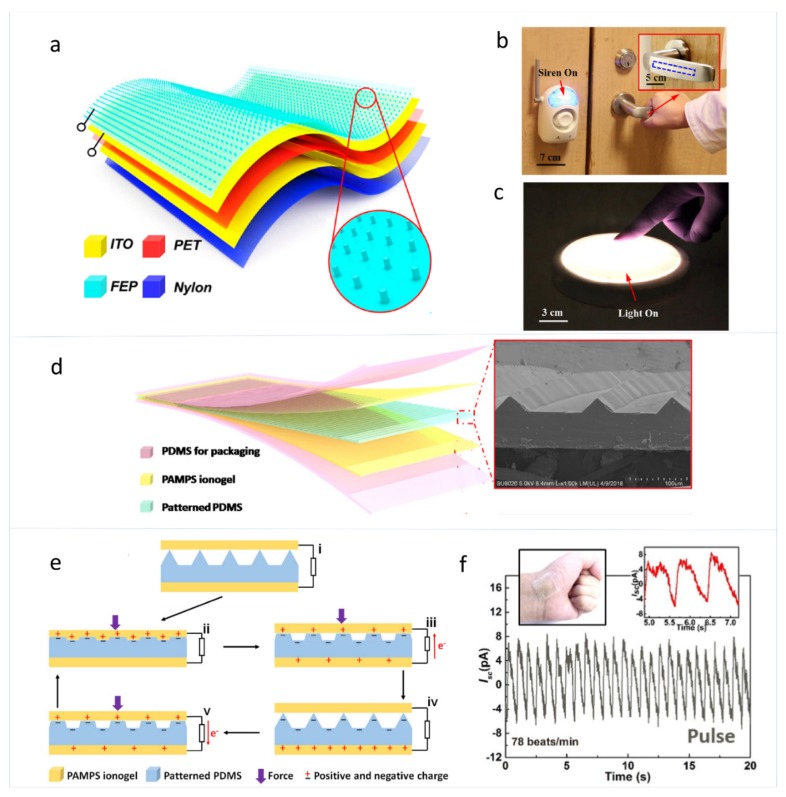
(**a**) Schematic illustration of the structural design of triboelectric sensor (TES). (**b**) Triggering a wireless alarm system by grasping the TES that is installed on a door handle. (**c**) Switching a panel light by a finger touching the TES that is applied on the surface of the light [37]. Copyright 2014, American Chemical Society. (**d**) Sketch of the layered structure of the sensor and SEM image of the patterned PDMS film with protruding triangular stripes. (**e**) A complete working cycle of the ionogel-based TENG sensor. (**f**) The output current of the sensor generated by human pulse beats [71]. Copyright 2016, Wiley.

Dielectric materials with strong electron affinity are usually used as the negatively charged triboelectric layer for skin TENGs used for touch/pressure sensors working in the single-electrode mode [37,67,68,69,70]. Zhu et al. reported a TENG-based flexible tactile sensor which has a polymer-nanowire modified surface, as shown in Figure 4a–c [37]. As shown in Figure 4a, a layer of polyethylene terephthalate (PET) is sandwiched between two ITO electrodes, and a layer of fluorinated ethylene propylene (FEP) is applied as an electrification layer, which is modified by vertically aligned polymer nanowires (PNWs) on the surface. In the extremely low-pressure region (<0.15 kPa), this self-powered touch and pressure sensor which relies on contact electrification to generate voltage signals shows a pressure sensitivity of $44{\;\mathrm{mV}\;\mathrm{Pa}}^{-1}$ ($0.09{\%\;\mathrm{Pa}}^{-1}$) and a maximum touch sensitivity of $1.1{\;V\;\mathrm{Pa}}^{-1}$ ($2.3{\%\;\mathrm{Pa}}^{-1}$).

For skin TENGs to detect touch/pressure working in the attached-electrode mode, an air gap between the two triboelectric layers is necessary so that the triboelectric charges on the two surfaces can contact and separate, which leads to electrical outputs [18,71,72,73,74]. Zhao et al. reported a skin TENG based self-powered tactile sensor with good pressure and tactile sensing performance, which has a maximum sensitivity of $1.76{\;V\;N}^{-1}$, as shown in Figure 4d–f [71]. The skin TENG has a double network ionogel and patterned polydimethylsiloxane (PDMS) with a dihedral stripes structure as the two triboelectric layers. A complete working cycle of the skin TENG is illustrated in Figure 4e to show the working principle of the sensor. This transparent and stretchable self-powered sensor has wide applications as wearable electronics to detect touching forces of different magnitudes, finger bending, human breathing, and pulse beating.

### 3.2. Piezoelectric Nanogenerators as Self-Powered Touch/Pressure Skin Sensors

With the use of piezoelectric materials, PENGs can directly convert the change of pressure into the change of electrical signals, which makes them a very good choice for monitoring touch/pressure. The piezoelectric materials used in PENG-based self-powered touch/pressure skin sensors include inorganic and organic materials. The inorganic piezoelectric materials are mainly nanomaterials of ZnO and piezoelectric ceramics [75,76,77,78], while organic piezoelectric materials are mainly PVDF-based materials [38,79,80,81,82].

**Figure 5:**
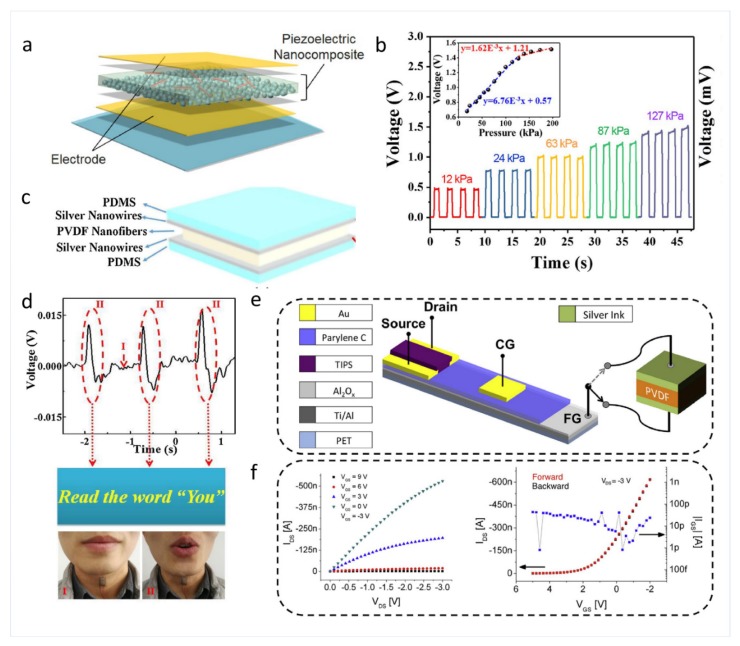
(**a**) The schematic illustration of the fabricated 0–3 carbon nanotubes (CNTs)-doped ceramic epoxy piezoelectric nanocomposite film. (**b**) The pressure response of the nanocomposite sensor with 0.07 wt% CNTs under pressure range from 0 to 200 kPa [75]. Copyright 2018, Elsevier. (**c**) Schematic diagram of pressure signal detection of the flexible pressure sensor. (**d**) Open-circuit voltage of the sensor when attached to the vocal cords [38]. Copyright 2018, Elsevier. (**e**) Structure and employed materials of the PVDF-based tactile sensor. (**f**) Output and input properties of the device for pressure sensing [79]. Copyright 2016, Elsevier.

The PENG-based self-powered touch/pressure skin sensor based on carbon nanotubes (CNTs)/piezoelectric ceramic composite have been reported to possess high sensitivity. For example, Kim et al. fabricated a flexible piezoelectric pressure skin sensor based on CNTs-doped 0–3 ceramic-epoxy nanocomposites, using ceramic powders and epoxy resin with the contents of 81 and 19 wt%, respectively, as shown in Figure 5a,b [75]. In this skin sensor, when the content of carbon nanotubes is 0.07 wt%, the piezoelectric coefficient of epoxy ceramic composite film reaches 68 pc/n. Additionally, it shows a linear response up to 150 kPa with a sensitivity of $6.76{\;\mathrm{mV}\;\mathrm{kPa}}^{-1}$ (67.6 mV/N). PENG-based self-powered touch/pressure skin sensors based on organic piezoelectric materials have also been reported. Wang et al. reported a PENG-based pressure skin sensor which consists of electrospinning-prepared PVDF nanofiber film with PDMS/Ag NWs and PET/ITO as the two electrodes, as shown in Figure 5c,d [38]. The PENG pressure skin sensor can capture tiny mechanical signals and have good flexibility and sensitivity. It also has the advantage of a simple preparation process. Spanu et al. reported a PENG-based tactile skin sensor based on an organic charge modulated FET coupled with a PVDF film, which is able to reliably transduce pressure as low as 300 Pa in a wide frequency range (20~500 Hz), as shown in Figure 5e,f [79].

To summarize this section, for TENG-based self-powered touch/pressure skin sensors, the two kinds of working modes have their own merits and drawbacks. Table 2 shows the comparison of self-powered skin sensors for detecting touch and pressure in terms of mechanism, materials, and sensitivity. Skin TENGs working in the single-electrode mode have simple manufacturing but are more easily to be impacted by the surrounding environmental situations. Skin TENGs working in the attached-electrode mode are less affected by the environment, but their sensing performance will be affected by the device deformation before applying the detected stimuli. For PENG-based self-powered touch/pressure skin sensors, in general, the reported inorganic piezoelectric materials such as ZnO and PZT have relatively high piezoelectric coefficients, but relatively low flexibility; while the reported piezoelectric polymers such as PVDF have relatively high flexibility, but relatively low piezoelectric coefficients. Piezoelectric pressure skin sensors have been widely used in the detection of heartbeat, pulse, slip, and other dynamic pressures due to their high sensitivity and fast response time.

## 4. Self-Powered Acoustic Skin Sensors

Acoustic sensors are devices that can accept sound waves and convert sound signals into electrical signals. Self-powered acoustic skin sensors do not need energy-supply components and can not only be used as microphones to capture sound in a noisy environment, but can also play an important role in the field of hearing aids [39,40,83]. Furthermore, acoustic sensors are the core components of artificial intelligence, and widely used in augmented reality (AR), virtual reality (VR), automation, and other fields [84,85]. Acoustic sounds are also a type of mechanical motion, and the reported self-powered acoustic skin sensors are also mainly based on TENGs and PENGs [86,87,88].

**Figure 6:**
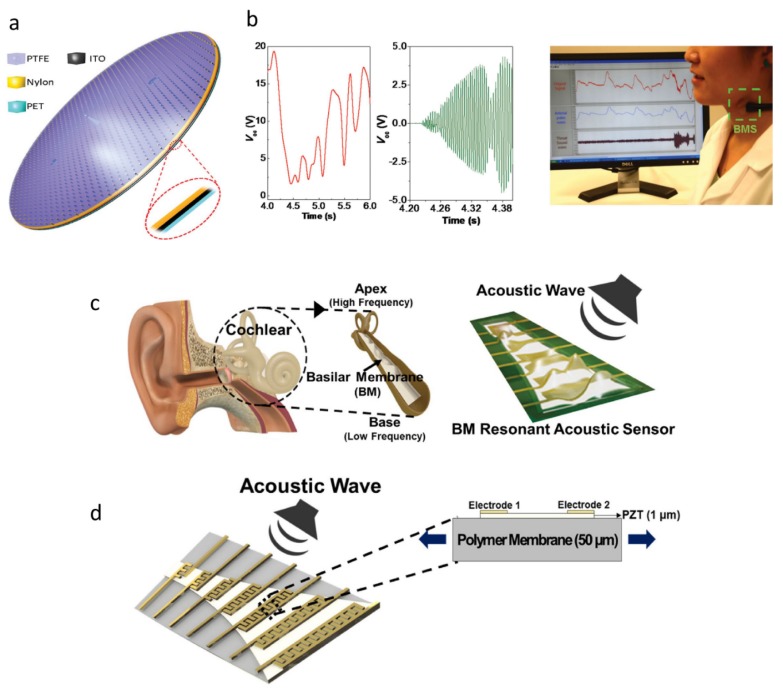
(**a**) Schematic diagram of the acoustic sensor. (**b**) Images showing the low-frequency component and high-frequency component of the output signals when the TENG is worn on the neck as a self-powered throat microphone [39]. Copyright 2015, Wiley. (**c**) Graphic image of the basilar membrane (BM) in the human cochlear which is naturally designed with an asymmetric trapezoidal shape and the sketch of the multi-channel piezoelectric acoustic sensor inspired by the resonant structure of the BM. (**d**) Schematic diagram of the sensor in response to the sound wave and the enlarged cross-sectional image of the dashed acoustic sensor part [40]. Copyright 2018, Elsevier.

Yang et al. reported a TENG-based self-powered acoustic skin sensor, which can pick up and recover human throat voice even in an extremely noisy or windy environment. This sensor consists of a nylon thin film coated by ITO as one electrification layer, a PTFE layer as the other electrification layer, and an oval shape layer of PET acting as the supporting substrate, as shown in Figure 6a,b [39]. The working principle can be elucidated as that the sound waves promote the contact/separation between PTFE and nylon, which results in electrical signals. The acoustic skin sensor has a sensitivity of $51\;{\mathrm{mV}\;\mathrm{Pa}}^{-1}$, a response time of less than 6 ms, a pressure detection limit down to 2.5 Pa, and a wide frequency range from 0.1 to 3.2 kHz. Han et al. reported a flexible basilar membrane-inspired PENG-based acoustic skin sensor in the human cochlear, which contains a PZT thin film with good piezoelectric effect and multi-channels composed of seven interdigitated electrodes, as shown in Figure 6c,d [40]. The acoustic skin sensor has an asymmetric trapezoidal structure and generates enough output voltage through the unique resonance motion of the PZT film. Through the combination of low quality factors between 18 and 28 and multi-resonance frequency tuning, this acoustic sensor can cover the voice frequency ranging from 100 to 4000 Hz and exhibit a sensitivity four to eight times higher than the conventional condenser sensor.

These self-powered acoustic skin sensors mainly use the vibration of sound waves to trigger electrical outputs. Up to now, the studies on self-powered acoustic skin sensors have not been as numerous as those on self-powered body motion and touch/pressure skin sensors. However, the important position in hearing aids may promote the research and development of self-powered acoustic skin sensors in the future.

## 5. Other Kinds of Self-Powered Skin Sensors

The self-powered skin sensors mentioned above are all related to mechanical motion, which are mainly based on mechanical energy harvesters, TENGs, and PENGs. Besides, there are also some self-powered skin sensors that are not related to mechanical motion. They are usually used to monitor body temperature and chemicals in sweat [89,90,91]. In daily life, body temperature and sweat sensors are widely used in areas such as healthcare and sports.

**Figure 7:**
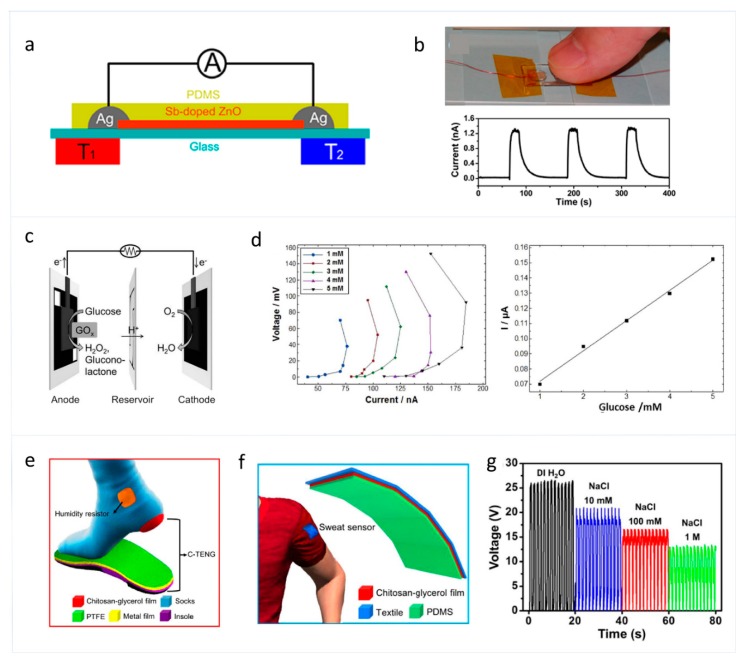
(**a**) Schematic diagram of a single Sb-doped ZnO microbelt nanogenerator. (**b**) Optical image showing a finger touching an electrode of the nanogenerator and its corresponding output current [46]. Copyright 2012, American Chemical Society. (**c**) Schematic illustration of the structure of the biofuel cell. (**d**) Diagrams of polarization curves with different concentrations of glucose, and calibration curve of output current versus the concentration of glucose in mM with a 1 MΩ resistor connecting the device’s electrical leads [47]. Copyright 2018, Elsevier. (**e**) The schematic illustration of the TENG connected on socks. (**f**) Schematic structure of the self-powered sweat sensor on commercial fabrics. (**g**) Output voltage of the sweat sensor when detecting clothes adsorbed with NaCl solution with various concentrations [48]. Copyright 2018, Elsevier.

Self-powered temperature skin sensors are used to continuously monitor temperature changes in the human body, which are mainly based on thermoelectric nanogenerators [46,92,93]. These kind of skin sensors have important applications in sports and medical treatment. Yang et al. reported a thermoelectric nanogenerator based on single Sb-doped ZnO micro/nanobelts as a self-powered temperature sensor which has a reset time of about 9 s, as shown in Figure 7a,b [46]. The thermoelectric-nanogenerator-based skin sensor is structured with a single Sb-doped ZnO microbelt with an end attached to a glass substrate, which has an output voltage of 10 mV and an output current of 194 nA. Additionally, the single Sb-doped ZnO microbelt shows a Seebeck coefficient of about $350{\;\mu V\;K}^{-1}$ and a power factor of about $3.2\times 10^{4}{\;W\;\mathrm{mK}}^{-2}$.

As for sweat sensing, biofuel-based self-powered sweat skin sensors can be used for directional monitoring of a biological molecule in the sweat [47,94,95]. For example, Christopher et al. reported a paper-based self-powered sweat skin sensor based on glucose/oxygen enzymatic fuel cells for glucose monitoring in sweat, which shows a linear range of output current at 1~5 mM glucose ($R^{2}=0.996$) with a sensitivity of $0.02{\;\mu A\;\mathrm{mM}}^{-1}$, as shown in Figure 7c,d [47]. This glucose skin sensor has low cost, good selectivity, and rapid response to glucose with no interference from other biological molecules. The glucose sensor has great potential for applications in the diagnosis of diabetes. Furthermore, skin TENGs have also been applied in self-powered sweat sensing. Jao et al. reported a skin TENG consisting of a chitosan-glycerol film which has environmental stability, biocompatibility, adaptability, and flexibility, as shown in Figure 7e,f [48]. When working as a self-powered sweat sensor, the concentration of sodium chloride (NaCl) in human sweat can be diagnosed through the electrical signals generated by the contact and separation between the PDMS and sweat, the magnitudes of which change in response to different NaCl concentrations.

## 6. Summary and Prospects

In this review, the recent advances in self-powered skin sensors have been comprehensively summarized and discussed. The self-powered skin sensors do not need any energy-supply components like batteries; therefore, they can work continuously and are free from the trouble of replacement of batteries. Compared with non-self-powered skin sensors, self-powered skin sensors have their unique advantages but also face great challenges. Besides the merit of working without energy-supply components, self-powered skin sensors for body motion and touch/pressure detection can achieve real-time sensing and possess fast response time as well as long cyclic lifetime, which are advantageous compared with those non-self-powered sensors that have undesirable hysteresis in response and limited cyclic lifetime. In addition, the performance of the self-powered skin sensors is comparable to that of the non-self-powered sensors in terms of sensitivity [89,96,97,98,99]. However, the reported self-powered body motion skin sensors are mostly used for only qualitative detection and lack the ability of accurate quantitative detection. Future efforts may be needed to introduce a reliable calibration system for self-powered body motion skin sensors in order to achieve accurate quantitative measurements. Further improving the performance of the self-powered skin sensors including sensitivity and reliability is also required. Moreover, although the self-powered skin sensor itself does not need energy-supply components, its data processing terminal still needs external power supply. To achieve fully self-powered skin sensing with both self-powered detection and signal processing, integrating the self-powered skin sensor with energy-harvesting and energy-storage components to form a self-charging skin sensing system may be a possible solution [100,101,102]. Up to now, the self-powered skin sensors have been mainly based on energy harvesters, with their electrical signals stimulated by the detected source. Most of the reported self-powered skin sensors are motion-related and are mainly used for monitoring body motion, pressure/touch, and voice. These motion-related self-powered skin sensors are generally based on two kinds of mechanical energy harvesters, PENGs and TENGs. TENGs as self-powered skin sensors have the advantages of simple structure, easy fabrication, multiple choice of materials, and low cost; however, they are susceptible to environmental factors such as humidity. PENGs as self-powered skin sensors have the merits of high sensitivity and reliability. However, the key materials used are limited to piezoelectric materials. What is more, the piezoelectric materials with higher piezoelectric coefficients usually have poorer flexibility, while the piezoelectric materials with better flexibility usually have lower piezoelectric coefficients. Seeking breakthroughs in a variety of piezoelectric materials is one of the biggest challenges PENGs face. Besides motion-related self-powered skin sensors, there are self-powered skin sensors that are non-motion related, including self-powered temperature skin sensors and self-powered sweat sensors. Self-powered temperature skin sensors are used to detect body temperature and are mainly based on thermoelectric nanogenerators. Efforts such as developing new thermoelectric materials and optimizing the device structures are required to further improve the sensitivity and conformability of such self-powered temperature skin sensors. Self-powered sweat sensors are used to detect chemicals in sweat such as glucose and lactate, which are mainly based on biofuel cells. Enzymatic biofuel cells are the most reported self-powered sweat skin sensors because of their advantages of high catalytic activity, relatively low cost, and biocompatibility. However, they face challenges such as short lifetimes, reduced performance resulting from slow direct electron transfer, and temperature impacted activity and stability of the enzymes.

Overall, self-powered skin sensors have tremendous potential for applications in myriad areas, such as medical care, human biology, robotics, prosthetics, and sports; but in the meantime, they face lots of challenges. From the perspective of materials, new materials may need to be developed to simultaneously meet the requirements including adaptivity, air permeability, and reliability. From the perspective of the device, efforts are still required to improve the sensitivity, enhance the accuracy, and reduce the impact of environmental noise. From the perspective of commercialization, new fabrication techniques may be required in order to achieve large-scale production. It is predicted that with the fast development of materials and technologies, self-powered skin sensors will have a broad market and bright future.

## Figures and Tables

**Table 1:** Comparison of self-powered skin sensors for detecting body motion.

Mechanism	Material	Motion Detected	Output Power Density	Reference
TENG	Rubber and Al	Diaphragmatic breathing and joint movements	$76.27{\;W\;m}^{-2}$	[35]
TENG	Rubber and physiological saline	Swallowing, calf raising, jumping, squatting, breathing, bicep curling, and gait patterns		[49]
TENG	PDMS and Au nanosheets film	Bending and relaxation of joints		[50]
TENG	PEDOT:PSS coated textile and PTFE	Hand motion and finger bending	$2{\;W\;m}^{-2}$	[51]
TENG	Kapton and Cu	Motion of joints and muscles	$5{\;W\;m}^{-2}$	[52]
TENG	PTFE and Nylon	Respiration		[56]
PENG	ZnO	Eye ball motion		[36]
PENG	ZnO and PVDF	Joint bending		[53]
PENG	PZT	Joint posture	$3.93{\;\mu W\;\mathrm{cm}}^{-3}$	[60]
PENG	BN nanosheets	Movements	$106{\;\mu W\;\mathrm{cm}}^{-3}$	[63]

**Table 2:** Comparison of self-powered skin sensors for detecting touch and pressure.

Mechanism	Materials	Sensitivity	Reference
TENG	FEP and ITO	Pressure sensitivity of $44{\;\mathrm{mV}\;\mathrm{Pa}}^{-1}$ and maximum touch sensitivity of $1.1{\;V\;\mathrm{Pa}}^{-1}$	[37]
TENG	Silicone rubber and silver-coated nylon yarn		[69]
TENG	PDMS and ITO	$0.29{V\;\mathrm{kPa}}^{-1}$	[67]
TENG	PAMPS ionogel and PDMS	$1.76{\;V\;N}^{-1}$	[71]
TENG	Cellulose nanofibril composite and PI aerogel		[74]
PENG	CNTs-doped 0–3 ceramic-epoxy nanocomposites	$6.76{\;\mathrm{mV}\;\mathrm{kPa}}^{-1}$	[75]
PENG	PVDF nanofibers		[38]
PENG	ZnO homojunction nanowire		[78]
PENG	FET and PVDF		[79]
